# Circulating vitamin D level before initiating chemotherapy impacts on the time-to-outcome in metastatic colorectal cancer patients: systematic review and meta-analysis

**DOI:** 10.1186/s12967-024-04889-2

**Published:** 2024-01-30

**Authors:** Alessandro Ottaiano, Maria Lucia Iacovino, Mariachiara Santorsola, Sergio Facchini, Domenico Iervolino, Francesco Perri, Guglielmo Nasti, Vincenzo Quagliariello, Nicola Maurea, Andrea Ronchi, Bianca Arianna Facchini, Alessia Bignucolo, Massimiliano Berretta

**Affiliations:** 1 Istituto Nazionale Tumori di Napoli, IRCCS “G. Pascale”, 80131 Naples, Italy; 2 Division of Medical Oncology, Department of Precision Medicine, University of Campania Luigi Vanvitelli, 80138 Naples, Italy; 3 Division of Cardiology, IRCCS “G. Pascale”, 80131 Naples, Italy; 4 Pathology Unit, Department of Mental Health and Physic and Preventive Medicine, University of Campania Luigi Vanvitelli, Naples, Italy; 5 Integrative Medicine Research Group (IMRG), Noceto, 43015 Parma, Italy; 6 Department of Clinical and Experimental Medicine, University of Messina, 98122 Messina, Italy

**Keywords:** Metastatic colorectal cancer, Vitamin D, Prognosis, Meta-analysis, Overall survival, Progression-free survival

## Abstract

**Background:**

Vitamin D (VD) is implicated in various health conditions, including colorectal cancer (CRC). To investigate potential relationships between pre-chemotherapy VD levels and the time-to-outcome in metastatic CRC patients, we conducted a systematic review and meta-analysis.

**Methods:**

Following the PRISMA 2020 guidelines, we performed thorough searches in PubMed/MEDLINE and Scopus/ELSEVIER databases (covering the years 2002 to 2022). Inclusion criteria mandated studies to report on individuals aged 18 years and above with histologically confirmed stage IV CRC. Additionally, studies needed to provide data on VD levels before chemotherapy, along with hazard ratios (HR) and 95% confidence intervals (CIs) for overall survival (OS) and/or progression-free survival (PFS). Five articles were identified with the aim of establishing a combined risk estimate for death and progression based on pre-chemotherapy VD levels. Heterogeneity among studies and publication bias were evaluated using Tau^2^, I^2^ statistics, and a Funnel plot.

**Results:**

Although no significant heterogeneity was observed in time-to-outcome among the selected studies, variations in technical assessments and serum VD concentration measurements were noted. The pooled analysis, involving 1712 patients for OS and 1264 patients for PFS, revealed a 47% increased risk of death (HR: 1.47, 95% CI: 1.21–1.79) and a 38% increased risk of progression (HR: 1.38, 95% CI: 1.13–1.70) for patients with lower VD levels, as indicated by fixed-effects models.

**Conclusions:**

Our results emphasize the adverse effects of low VD concentration on the time-to-outcome in metastatic CRC patients. This underscores the importance of investigating VD supplementation as an innovative approach in this clinical setting to enhance patient outcomes.

**Supplementary Information:**

The online version contains supplementary material available at 10.1186/s12967-024-04889-2.

## Introduction

Colorectal cancer (CRC) is a highly prevalent malignancy worldwide, ranking as the third most commonly diagnosed neoplasm with approximately 1,931,590 new cases annually [1]. At the time of diagnosis, nearly 30% of CRC cases are already identified as stage IV, metastatic disease [2]. The liver is the most frequently involved metastatic site, followed by lymph nodes and lungs. Standard treatment options for this stage, guided by molecular assessments (RAS and BRAF mutational status, HER2 amplification), predominantly involve systemic therapies. These therapies comprise chemotherapy, either as single agents (fluorouracil, tipiracil/trifluridine, oxaliplatin, irinotecan) or in combination with monoclonal antibodies (bevacizumab, panitumumab, cetuximab, trastuzumab). In refractory cases, regorafenib, a multi-kinase inhibitor, can be administered orally as monotherapy. Recently, the combination of encorafenib, an oral inhibitor targeting the BRAF p.V600E mutation, with cetuximab, has emerged as a second-line therapy. Immunotherapy, although applicable to a small fraction of patients with microsatellite instability (< 5%), has demonstrated limited efficacy. Despite notable advancements, the overall survival for patients with metastatic CRC seldom exceeds 30 months [3, 4].

Cancer is a complex disease where the integration of both genetic and epigenetic factors collaborates to influence biological and clinical trajectories [5–7]. Prediction of prognosis in metastatic CRC is of paramount importance, as the identification of clinical and/or biological factors that can modify it may contribute to better patient stratification and guide the selection of more intensive therapies or monitoring approaches. Furthermore, understanding these factors can provide insights into the underlying biology of cancer and facilitate the identification of innovative therapeutic targets. Several clinical and biological factors are utilized for both clinical-prognostic stratification and therapeutic targeting in metastatic CRC, including initial tumor burden, CEA levels, performance status, BRAF and RAS mutations, and microsatellite status [8].

Among the substances that have garnered significant interest for their unexpected involvement in cancer is vitamin D. Vitamin D3 (abbreviated as VD) is a fat-soluble molecule derived from 7-dehydrocholesterol and activated by ultraviolet light in the skin [9]. Hence, the methodological need, not always met, to specify the season when assessing VD levels. Once VD enters the bloodstream, it undergoes hepatic conversion by the enzyme 25-hydroxylase, resulting in the formation of 25-hydroxyvitamin D [25(OH)D], also known as calcidiol. This is the primary circulating form of VD and is commonly measured to evaluate individual levels. Subsequently, 25(OH)D is transported to the kidneys, where it undergoes a second hydroxylation step by the enzyme 1α-hydroxylase to form 1,25(OH)2D, also known as calcitriol. Calcitriol binds to various cytosolic receptors and exerts its effects by translocating to the nucleus, thereby modulating gene expression [10]. This reprogramming leads to the regulation of calcium homeostasis. While VD plays a critical role in calcium absorption and bone resorption [11], it is also implicated in other clinical conditions such as cancer, depression and cardiovascular diseases [12–16]^.^

Recent meta-analyses have shown a positive association between VD and cancer-specific survival in CRC patients. However, these studies primarily focused on CRC susceptibility or included patients at all stages of the disease without distinguishing the prognostic impact of low versus high VD categories specifically in metastatic stage IV CRC patients [17–22].

In this systematic review and meta-analysis, we aim to evaluate the relationship between VD levels and time-to-outcome in stage IV CRC patients. Consequently, we present a pooled and updated estimation of the risk of death and disease progression in stage IV CRC patients based on VD levels.

## Methods

This article presents a comprehensive review and meta-analysis that investigates the correlation between circulating VD [represented as 25(OH)D] levels prior to initiating first-line chemotherapy and survival outcomes among individuals diagnosed with stage IV (metastatic) CRC. The study adhered to the PRISMA statement of 2020, and a meticulously designed protocol registered in PROSPERO (ID438513) was established from the outset. The selection criteria and methodologies were explicitly outlined within this protocol.

### Selection criteria

A comprehensive hand-search was conducted in two prominent international paper databases, namely PubMed/MEDLINE and Scopus/ELSEVIER, to identify relevant studies related to CRC. A manual searching method, designed to leverage the inherent advantages of a human-driven search strategy and ensure a nuanced and context-aware inclusion process, was implemented. Specifically, six authors were organized into two independent teams of three members each to carry out the manual search (Team 1: A.O., M.L.I., M.B., and Team 2: S.F., B.A.F., D.I.). The initial search was performed by Team 1, and the second was replicated by Team 2. This collaborative initiative aimed to leverage the diverse expertise and perspectives of team members, enhancing the sensitivity of the search process. Despite being time-consuming, the manual approach facilitated a deep comprehension of the scientific context, ensuring the inclusion of studies that might escape automated tools. The human-driven search strategy allowed for dynamic adaptation of search parameters, accommodating evolving insights during the review process. Moreover, manual selection enabled a nuanced evaluation of study quality, considering methodological intricacies that automated tools might overlook. Each team member independently explored relevant databases, pertinent journals, and additional sources to compile a comprehensive dataset. To uphold the rigor of the selection process, regular consensus discussions involving all six authors provided a platform for resolving occasional discrepancies.

The search strategy employed the following keywords: "colorectal cancer" OR "colon" OR "rectal" AND "vitamin D" OR "cholecalciferol" OR "calcidiol". The search encompassed articles published between 2002 and 2022 (last accessed on July 31st, 2022). PubMed/MEDLINE and Scopus/ELSEVIER were selected as primary databases for our meta-analysis due to their widely acknowledged extensive coverage of biomedical literature. PubMed is esteemed for its thorough coverage of medical literature, rendering it an indispensable resource for oncology-related studies. Complementing this, Scopus, with its multidisciplinary approach, provides a comprehensive array of scholarly articles, ensuring a thorough exploration of the prognostic implications of VD in metastatic CRC. The chosen time range from 2002 to 2022 aligns with our objective to identify comprehensive and pertinent studies. This timeframe resulted from consensus discussions among authors, considering methodological robustness, study quality, and technological advancements. Focusing on this period allows us to capture recent advancements in CRC research while maintaining a substantial historical context. This approach enhances the reliability and relevance of our findings, ensuring a nuanced understanding of the prognostic significance of VD in metastatic CRC.

To ensure the inclusion of suitable studies, a set of predetermined criteria was applied. Articles written in English were selected to mitigate language and publication biases. The target population comprised individuals aged 18 years and above. Only histologically confirmed stage IV CRC cases were considered and only studies that reported VD levels measured before chemotherapy were included. Furthermore, studies needed to explicitly provide hazard ratios (HR), along with corresponding 95% confidence intervals (CIs), for overall survival (OS) and/or progression-free survival (PFS) specifically calculated for stage IV CRC patients. The information regarding HR was sought in all sections of the article, including supplementary files. No restrictions were imposed on the type of VD supplementation, study design, or specific chemotherapy regimen utilized. Preclinical articles focusing on in vitro and/or animal experiments were excluded. Studies that evaluated VD in conjunction with other biomarkers were included only if VD was examined independently.

**Fig. 1:**
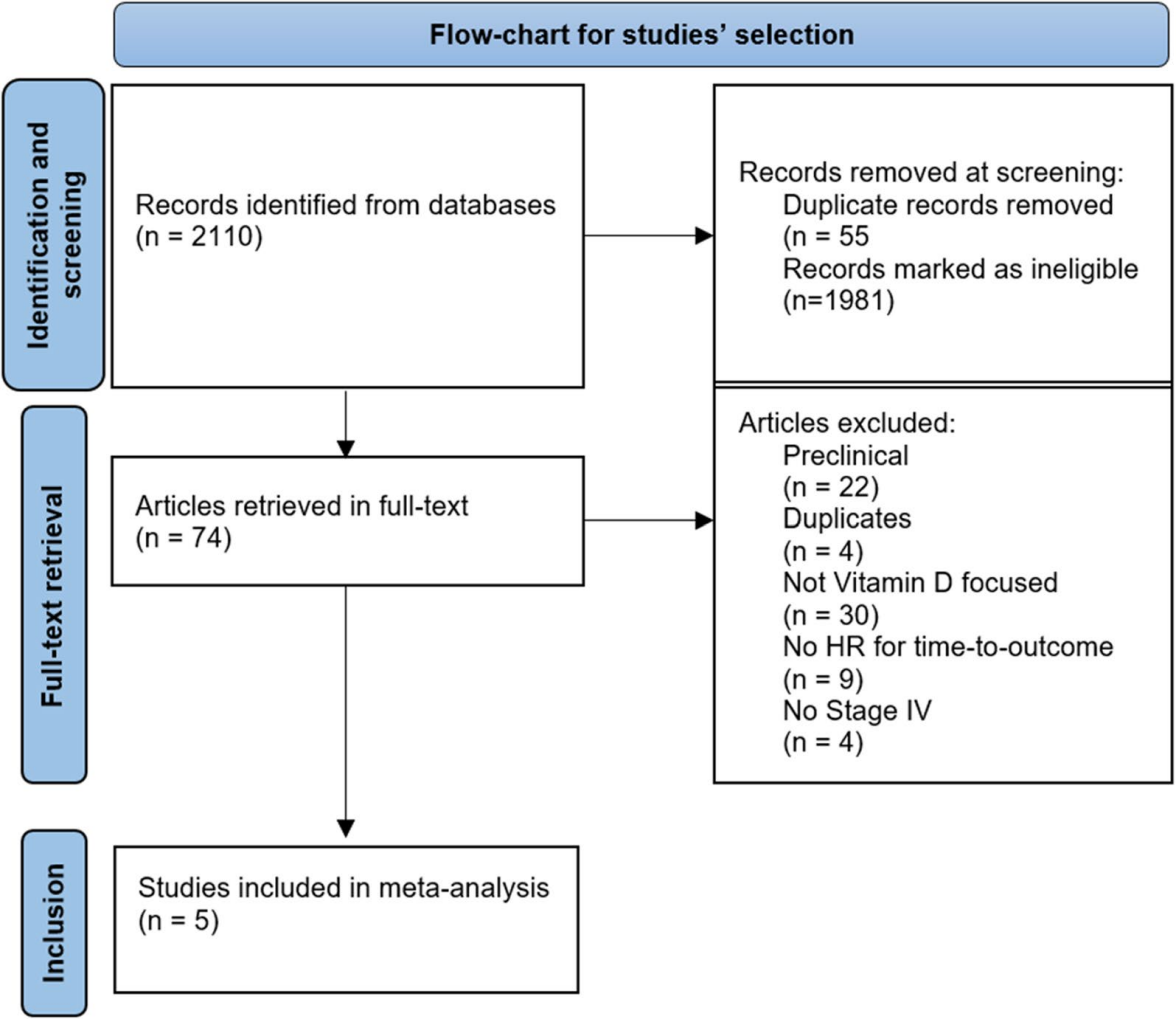
Flow-chart for studies’ selection

The inclusion criteria were meticulously formulated to ensure the relevance and quality of the selected studies, and some of them warrant detailed description. The choice of the English language helps mitigate language and publication biases, thereby enhancing the comprehensiveness of the literature. Specifying individuals aged 18 years and above and limiting the study population to histologically confirmed stage IV CRC cases maintains homogeneity in our analysis. Requiring measurement of VD levels before chemotherapy ensures a consistent baseline. The insistence on explicit reporting of HRs with corresponding CIs for OS and/or PFS ensures robust statistical evidence. The decision to exclude preclinical articles stems from the intention to maintain a clinical focus, prioritizing findings directly relevant to metastatic CRC patients. Preclinical studies, often conducted in vitro or on animal models, may introduce variables not directly applicable to human patients, thus enhancing the robustness of our analysis by concentrating on clinically pertinent outcomes. Furthermore, the insistence on independently examining VD from other biomarkers is rooted in the need for clarity in prognostic attribution. Including additional biomarkers alongside VD could potentially confound interpretations, making it challenging to discern whether observed prognostic effects are solely attributable to VD. By isolating VD in our analysis, we aim to provide a clear and unambiguous understanding of its distinct prognostic significance in the context of metastatic CRC, avoiding potential confounding influences from other biomarkers. This approach strengthens the reliability and specificity of our findings. Please refer to Fig. 1 for a detailed flowchart illustrating the selection process.

### Data extraction

We extracted the following information for each study: primary author, publication year, study design, clinical-pathological characteristics of patients, methodology used for VD assessment, number of enrolled patients, duration of follow-up, and time-to-outcome data including hazard ratio (HR) and 95% confidence interval (CI). We specifically extracted the maximally adjusted HR from each study to minimize the impact of confounding effects. A team of three investigators (M.S., G.N., S.F.,) independently reviewed all the data. Any disagreements or concerns were addressed through discussions involving all authors.

### Primary endpoint

The main objective of this meta-analysis was to estimate the overall risk of death and disease progression in metastatic CRC patients based on their VD levels.

### Quality assessment

To ensure a comprehensive evaluation of quality and potential bias, four authors (A.O., M.L.I., M.S., and M.B.) were responsible for assessing the methodologies and results of the selected studies. The MINORS (Methodological Index for Non-Randomized Studies) criteria [23] and NOS (Newcastle–Ottawa Scale) [24] were employed to evaluate non-randomized trials. For randomized trials, the RoB2 (Risk of Bias 2) scale was utilized [25]. Independent rating of the final scores was performed by N.M. and B.A.F, who were blinded to the previous results. Any discrepancies were resolved through consensus discussions involving all authors. The distinct features and considerations that set apart the MINORS, NOS, and RoB2 scales are detailed in Additional file 1.

### Statistical methods

We performed a meta-analysis to investigate the association between VD levels and time-to-outcome in stage IV CRC patients. To account for low heterogeneity among the studies, a fixed-effects model was employed. Forest plots were used to present the results, displaying hazard ratios (HRs) and corresponding 95% confidence intervals (CIs), along with a final pooled HR. An HR of 1.0 indicates equal event probability (EP) in both low-VD and high-VD level groups (EP: VD low/EP VD high). Conversely, an HR greater than 1.0 suggests an increased risk of death or disease progression in the low VD level group. When HRs reported high VD levels in the numerator (VD high vs. low), the HRs and CIs were recalculated based on Altman et al. [26] to maintain consistency in the comparison trajectory between VD low vs. VD high (calculated HR VD low vs. high = 1/HR VD high vs. low), enhancing reader comprehension. Heterogeneity among the studies was assessed using I^2^ and Tau^2^ statistics [27]. I^2^ determines the proportion of observed variation attributed to true differences rather than chance. I^2^ = 100% × (Q − DF)/Q, where Q represents Cochran's heterogeneity statistic and DF represents degrees of freedom. Negative I^2^ values were set to zero, ensuring I^2^ falls between 0 and 100%. A value of 0% indicates no observed heterogeneity, while higher values indicate increasing heterogeneity. For example, an I^2^ of 0% indicates no heterogeneity, while 0–25% suggests low heterogeneity (variation among studies likely due to chance), > 25–50% moderate heterogeneity (considerable variability among studies, but may not substantially impact result interpretation), and > 50–75% high heterogeneity (significant variability among studies that may affect the reliability of the meta-analysis), > 75% considerable heterogeneity (meta-analysis results should be interpreted cautiously due to substantial heterogeneity likely impacting overall findings). In addition to I^2^, Tau^2^, accounting for study size, was employed to measure heterogeneity in this meta-analysis. Unlike I^2^, providing a relative measure, Tau^2^ estimates the absolute magnitude of between-study variance. The formula for Tau^2^ is Tau^2^ = (Q—DF)/[(k—1) × sum of inverse variances], where k is the number of studies included. Tau^2^ is particularly useful with varying sample sizes and effect estimates, reflecting true effect size variability. Larger Tau^2^ values indicate increased dispersion, emphasizing study-specific characteristics' influence. Values from zero to 0.1 indicate most variability due to random error rather than systematic differences, > 0.1 to 0.2 suggest moderate true between-study variance, indicating a moderate level of diversity in true effects among studies. Values above 0.2 indicate high true between-study variance and substantial variation in true effects, emphasizing study-specific characteristics' influence. In a hypothetical scenario, in a meta-analysis of cancer treatment efficacy, an I^2^ of 60% implies substantial variability in study outcomes beyond chance, prompting exploration of contributing factors. Simultaneously, a Tau^2^ of 0.25 highlights significant between-study variance, accentuating the need for cautious interpretation due to underlying diversity in treatment effects among studies. These statistics collectively guide researchers in interpreting meta-analytical outcomes.

Potential publication bias was assessed using a funnel plot [28]. The assessment of potential publication bias through funnel plots encompasses several crucial steps. Firstly, data are collected from selected studies, focusing on effect sizes such as HRs and their associated standard errors (SEs)**.** These are then used to construct a scatter plot, with effect sizes (HRs) plotted on the horizontal axis and precision (SEs) on the vertical axis. In an unbiased scenario, this plot would exhibit a symmetrical funnel shape. This implies that smaller studies, characterized by wider scattering, coexist with larger, more precise studies clustered near the top. Deviations from this expected symmetry signal potential issues, such as publication bias. Thus, the interpretation of these funnel plots involves a nuanced assessment of the degree and direction of any asymmetry observed. Factors like selective reporting or methodological variations could contribute to such irregularities. On a descriptive and intuitive perspective, the triangle symbolizes the expected distribution of studies in the absence of bias. Statistical tests, such as Egger's or Begg's, can be applied to formally document the asymmetry, providing a quantifiable measure of the likelihood of publication bias [28]. The significance of this process lies in its ability to bring attention to potential bias, offering researchers insights into whether positive or significant outcomes disproportionately influence the results. A symmetrical funnel plot enhances internal validity, suggesting that study findings are less likely influenced by publication bias.

Statistical analyses were conducted using MedCalc Statistical Software (MedCalc® Statistical Software version 19.6, MedCalc Software Ltd., Ostend, Belgium) and R studio software version 4.2.1 (R studio Inc. Company, Boston, MA, USA). MedCalc is renowned for its user-friendly interface and robust statistical capabilities, rendering it well-suited for applications in medical and clinical research. It was applied to perform the computation of descriptive statistics and the generation of databases. R Studio, developed on the foundation of the R programming language, permits extensive customization of statistical analyses. It was employed for intricate statistical procedures involving custom calculations, specialized algorithms or adjustments (such as the recalculations of Hazard Ratios), and the creation of high-quality graphics, exemplified by forest plots and funnel plots.

## Results

**Fig. 2:**
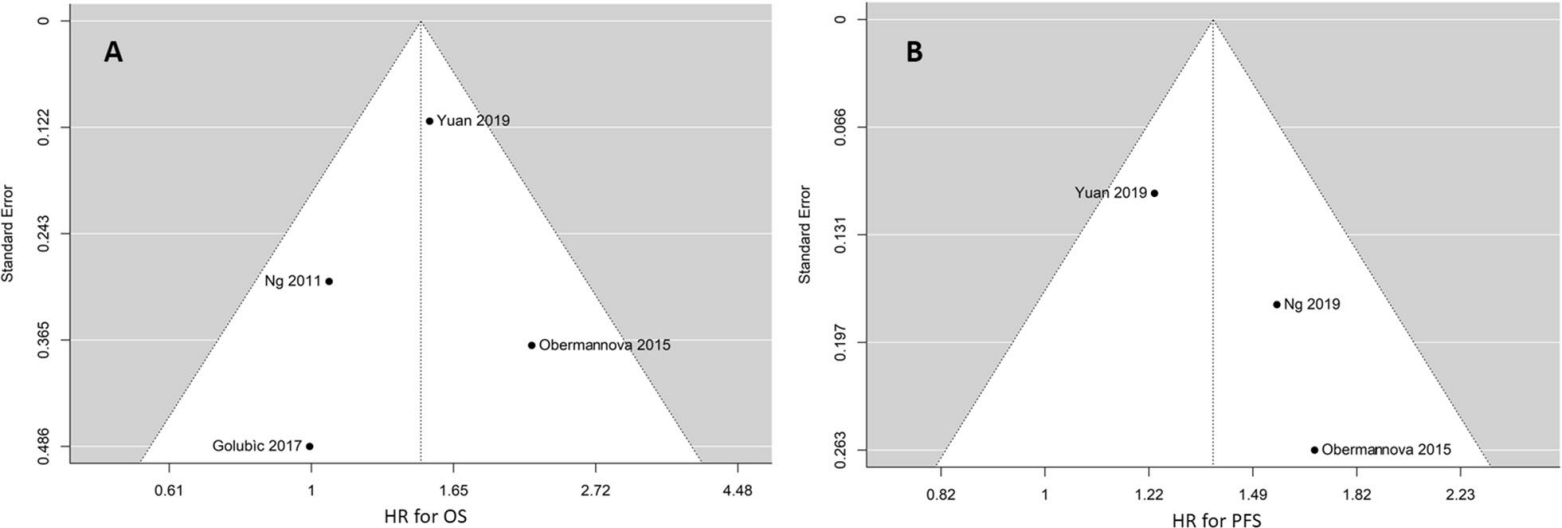
Funnel plots displaying the chosen studies for overall survival (OS) (**A**) and progression-free survival (PFS) (**B**). The x-axis represents the estimated outcome (HR: Hazard Ratio), while the y-axis typically indicates the standard error. No noticeable asymmetry is observed in the funnel plot, suggesting the absence of publication bias or small-study effects in this specific analysis

Five studies meeting the inclusion criteria were included in this meta-analysis [29–33]. The selection flowchart can be found in Fig. 1. There was no significant heterogeneity among the selected studies regarding overall survival (OS) and progression-free survival (PFS) (Tau^2^ values: 0.00% and 0.01%; I^2^ values: 0% and 22%, respectively). The funnel plots for both OS and PFS demonstrated a symmetrical distribution of the selected studies (Fig. 2A, B). Table 1 provides the MINORS, Newcastle–Ottawa Scale (NOS), and Risk of Bias 2 (RoB2) scores of the included articles.

**Table 1:** Study characteristics and quality

First author, year	Study design	Vitamin D assessment	Vitamin D adjusted per season	N. of patients	TTO	Comparison modalities	Other biomarkers evaluated	MINORS score	NOS score	RoB2 assessment
Ng, 2011	P	RIA	Yes	515	OS, PFS	Interquartile comparison, lowest ≤ 13.1 ng/mL *vs* highest ≥ 27.2 ng/mL)	No	7	7	NA
Obermannova, 2015	P	EBA	Yes	84	OS, PFS	Normal *vs* Low (cutoff: 40 nmol/L)	CEA	7	5	NA
Golubić, 2017	P	EBA	No	72	OS, PFS	No supplementation *vs* Supplementation (2000 UI/die) For two years Measurement in nmol/L*	No	NA	NA	Some concerns
Ng, 2019	P	RIA	No	139	OS, PFS	High dose supplementation (8000 IU/die for the cycle 1, then 4000 IU/die) *vs* Standard dose (400 UI/die) Chronic administration Measurement in ng/mL**	No	NA	NA	Low risk
Yuan, 2019	P	RIA	Yes	1041	OS, PFS	Interquintile comparison, lowest ≤ 10.8 ng/mL *vs* highest ≥ 24.1 ng/mL	No	7	6	NA

The table provides a summary of methodology and quality-related details from pertinent studies that explore the association between Vitamin D and clinical outcomes in metastatic colorectal cancer patients. The included studies are presented with the first author's name and publication year, followed by information on study design, methods of Vitamin D assessment, adjustment for Vitamin D levels per season, the number of patients involved, treatment outcomes, comparison modalities, other evaluated biomarkers, and assessment scores for MINORS, NOS, and RoB2

*%FAT* percentage body fat, *BMI* Body Mass Index, *CEA* Carcino-embryonic antigen, *EBA* electrochemiluminescence binding assay, *LA* locally advanced, *LC/MS* liquid chromatography-tandem mass spectrometry, *MINORS* Methodological Index for Non-Randomized Studies, *NA* not applicable, *NOS* Newcastle–Ottawa Scale, *OS* overall survival, *P* prospective, *PFS* progression free survival, *RIA* Radioimmunoassay, *RoB2* Risk-of-Bias v.2, *TTO* time-to-outcome

^*^No monitoring of VD levels was performed

^**^The VD levels after the first cycle of chemotherapy in high-dose VD group were constantly double compared to standard-dose group

A total of 1712 estimates for OS and 1264 estimates for PFS were included in the meta-analysis (Table 1). All studies were prospective and met the essential criterion of presenting independent hazard ratio (HR) analysis for stage IV CRC patients. Serum VD levels were determined through radioimmunoassay (RIA) or electrochemiluminescence binding assay (EBA). Three studies adjusted VD concentration based on the season, while two studies reported VD supplementation. Moreover, VD concentration was expressed using different measurement units, with three articles using [nmol/L] and two articles using [ng/mL]. The cutoff values for VD concentration (low vs. high) also varied. In a randomized study, although there was no monitoring of VD levels, the group without oral VD supplementation started with a median VD level of 35 nmol/L, while the group receiving oral supplementation started at 27 nmol/L.

Table 2 presents the clinical-pathological characteristics of the patients included in the selected articles. The cutoffs for age dichotomization into elderly and non-elderly patients varied and were only reported in two studies. Male gender was predominant, while the primary tumor side was unspecified in two articles.

**Table 2:** Clinico-pathological characteristics of patients included in the selected articles

Author	Year	Age			Gender		ECOG PS		Side	
		Young	Old	Median	Male	Female	0	≥ 1	Left	Right
Ng	2011	< 61: 244	≥ 61: 271	61	306	209	492	22	NR	NR
Obermannova	2015	≤ 65: 57	> 65: 27	62	49	35	15	69	NR	NR
Golubić	2017	NR	NR	69	37	35	NR	NR	47	24
Ng	2019	NR	NR	NR	79	60	69	70	92	47
Yuan	2019	NR	NR	NR	604	437	631	410	610	369

*ECOG* Eastern Cooperative Oncology Group, *NR* not reported, *PS* performance status

Table 3 provides the time-to-outcome estimates. Median follow-up duration was clearly appropriate considering the disease setting, and all studies reported this information.

**Table 3:** Detailed risk of death and progression in the selected articles

Author	Year	Median follow-up (months)	Median OS (months)	HR_OS_	CI_OS_	*P*_OS_	Median PFS (months)	HR_PFS_	CI_PFS_	*P*_DFS_
Ng	2011	61.2	NR	0.94	0.72–1.23	0.55	NR	NR	NR	NR
Obermannova	2015	24.2	41.2	2.220	1.074–4.592	0.031	15.4	1.699	1.016–2.845	0.043
Golubić	2017	46	39	1.0064	0.3882–2.609	0.989	10.5	NR	NR	NR
Ng	2019	22.9	24.3	NR	NR	NR	13	0.64	0–0.90	0.02
Yuan	2019	67.2	33	0.66	0.53–0.83	0.0009	11	0.81	0.66–1.00	0.03

The table presents a synthesis of clinical outcomes extracted from the selected studies. Each study is delineated by the first author's name and publication year, followed by pertinent information on median follow-up, median OS, HRs for OS, CIs for OS, p-values for OS, median PFS, HRs for PFS, CIs for PFS, and p-values for PFS

*CI* confidence interval, *HR* hazard ratio, *NR* not reported, *OS* overall survival, *P* p-value, *PFS* progression-free survival

**Fig. 3:**
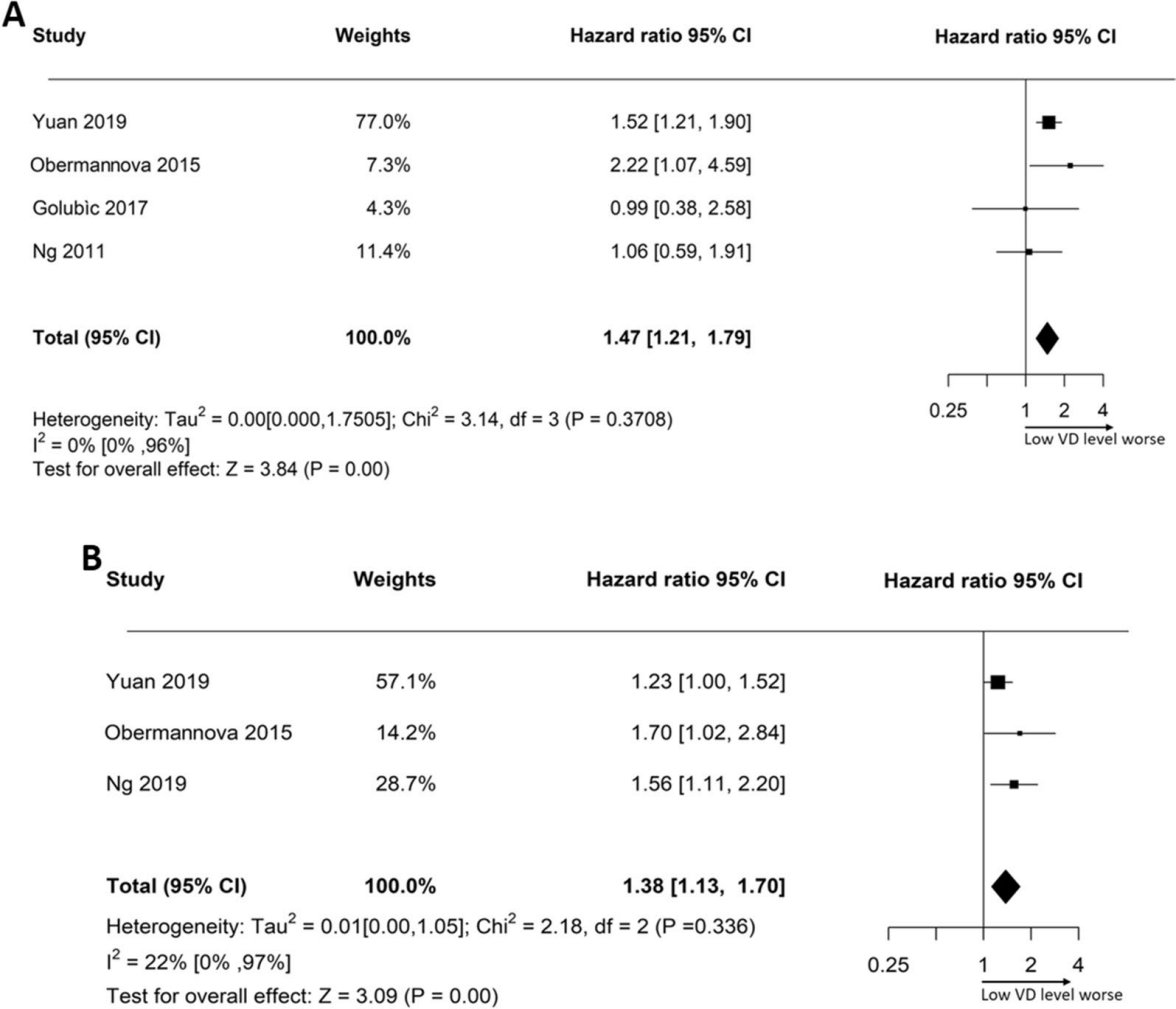
Plotting the forest graphs to illustrate the outcomes of overall survival (OS) (**A**) and progression-free survival (PFS) (**B**), stratified by varying levels of vitamin D (VD) in patients with metastatic colorectal cancer (CRC). The graphs present the Tau^2^, I^2^ statistics, and the combined hazard ratio (HR) using fixed-effects models

The effect size of VD levels on prognosis was determined by HR. Forest plots illustrating the effect of VD on OS and PFS can be seen in Fig. 3A and B, respectively. The pooled analysis showed a 47% increased risk of death in 1712 patients using fixed-effects models (HR: 1.47, 95% CI: 1.21–1.79). The pooled analysis for progression risk in 1264 patients resulted in a 38% increase (HR: 1.38; 95% CI: 1.13–1.70). Our findings suggest that inadequate VD concentration before initiating chemotherapy has a significant negative impact on OS and PFS in stage IV CRC patients.

## Discussion

Systemic treatments, such as chemotherapy combined with biological drugs, are globally recognized as the standard of care for patients diagnosed with stage IV CRC. The primary objective is to extend survival while maintaining an acceptable quality of life. Significantly, numerous studies have investigated the link between VD levels and CRC outcomes. However, these findings have shown some inconsistencies and lacked stratification based on stages or initial tumor burden [16–21]. In our study, we aimed to address this gap by specifically examining the influence of circulating VD levels on the prognosis of patients with metastatic CRC. By focusing on this specific patient population, our research holds clinical relevance in terms of assessing time-to-outcome. Furthermore, conducting a meta-analysis enables us to consolidate findings from multiple studies, providing a higher level of evidence and stronger conclusions.

Furthermore, it is essential to emphasize that the current landscape of prognostic tools and therapeutic approaches for metastatic CRC reveals significant gaps and challenges that demand attention. Existing models, relying on conventional clinical parameters (such as onset symptoms, tumor location, tumor burden, CEA level), pathological factors (histology, grading), and genetic molecular profiling (mutations in *RAS*, *BRAF*, *HER2* gene amplifications, etc.), may not adequately capture the dynamic and heterogeneous nature of metastatic CRC, thus limiting their precision in predicting individual patient outcomes. The description and discussion of current treatments in metastatic CRC are beyond the scope of this work. Nonetheless, therapeutic approaches, while advancing, still encounter challenges in achieving prolonged survival, particularly for patients with metastatic CRC. Therefore, a nuanced understanding of the molecular and biological intricacies of metastatic CRC, coupled with patient stratification, is crucial for optimizing treatment decisions. In this context, it is worth noting that there has been a recent surge in the literature examining the link between VD and cancer [34]. The active form of VD, 1,25-dihydroxyvitamin D, binds to the VD receptor (VDR) in various tissues, including the bone, intestine, and immune cells. VDR activation regulates calcium and phosphate metabolism, which are vital for maintaining skeletal health. Additionally, VDR activation influences cell differentiation and proliferation, and exhibits anti-inflammatory and anti-carcinogenic properties [35]. Recent research has suggested that VD exerts a protective effect against various cancer types. Mechanistically, VD demonstrates an anti-proliferative effect on cancer cells by inducing cell cycle arrest and apoptosis, and by reducing angiogenesis and metastasis [36]. Moreover, VD modulates the immune response, leading to the activation of natural killer cells and the inhibition of pro-inflammatory cytokines [37], which can positively impact chemotherapy efficacy and toxicity [38, 39]. Furthermore, a recent study indicates that VD may stimulate and support immune responses in CRC by modulating the function of T regulatory cells [40]. More recently, increasing evidence suggests that VD can influence the composition of the gut microbiota predominantly through immune system modulation, influence on intestinal barrier function, and that gut bacteria theyself can influence the metabolism of VD [41]. On the other hand, a complex relationships exists between gut microbial infections and cancer [42].

VD supplementation is an approach used to address and prevent VD deficiency, particularly for enhancing bone metabolism, on a global scale [43]. As previously mentioned, the activation of VDR plays a vital role in regulating calcium and phosphate metabolism, which is crucial for maintaining skeletal health. While a widely accepted consensus is still lacking, the recommended daily intake of VD varies depending on age and health condition. It can be acquired through dietary sources, such as fortified foods or supplements [44, 45]. Additionally, sufficient exposure to sunlight contributes to VD synthesis in the skin. VD supplements are available in various forms, including cholecalciferol (vitamin D3) and ergocalciferol (vitamin D2), and can be conveniently administered orally. Extensive research has also explored its involvement in other physiological functions, such as immune response and cellular proliferation [46]. Interestingly, two studies included in our meta-analysis employed VD supplementation as part of an interventional approach with regulated and/or controlled administration [31, 32]. However, information regarding whether VD intake was self-managed by patients or guided by healthcare professionals (through food and/or supplements) was lacking in the remaining studies. This heterogeneity poses a notable consideration in our meta-analysis.

Deficiency of VD can support CRC progression through multiple mechanisms. One of the key factors involves the anti-proliferative properties of VD. Research indicates that VD has the capacity to impede the growth and division of CRC cells [47]. Another intriguing aspect is the induction of apoptosis, facilitating the elimination of damaged or aberrant cells [48]. The anti-inflammatory properties of VD represent yet another indirect layer of defense. In fact, VD anti-inflammatory effects may contribute to a lowered risk of inflammation-related cancer progression [49]. The modulatory role of VD in the immune system adds another dimension to its potential protective effects. By enhancing immune function, VD may empower the immune mediated recognition and elimination of CRC cells [37]. Cell differentiation, a process regulated by VD, also emerges as a crucial factor. Proper cell differentiation is vital for maintaining healthy tissues and preventing the uncontrolled growth characteristic of CRC cells [50]. Furthermore, VD has demonstrated its ability to inhibit angiogenesis, the process through which tumors develop new blood vessels to sustain their growth. By impeding angiogenesis, VD may limit the blood supply to tumors, potentially impeding their progression [51, 52]. It is worth considering and discussing that, in three of the examined studies, the concentration of VD based on the seasons of blood collection (summer, autumn, winter, spring) was treated as a potentially conditioning variable in a multivariate analysis model for prognosis. Methodologically, it is crucial to emphasize that this approach is the most appropriate. Seasonal variations play a pivotal role in evaluating VD concentrations due to significant variability in sunlight exposure throughout the year. Heightened sunlight exposure during summer enhances cutaneous VD production, leading to elevated serum levels. Conversely, reduced sunlight exposure in winter may result in lower VD levels. Disregarding these seasonal variations could introduce confounding factors, compromising the accuracy of associations between VD levels and the prognosis of metastatic CRC. Our analysis of relevant studies demonstrates an increased risk of death and disease progression in metastatic CRC patients with low circulating VD levels before commencing chemotherapy, suggesting potential benefits of VD supplementation in this particular clinical scenario. Some limitations of our study warrant acknowledgment and discussion. Despite the inclusion of studies with sizable sample sizes, lengthy follow-up periods, and high-quality scores, methodological heterogeneity was observed in terms of VD assessment methods and cut-off values used to distinguish between low and high VD levels. Additionally, while the studies reported the administered chemotherapy regimens, they did not detail prognostic factors such as the initial disease burden, treatment responses, and toxicity. Furthermore, two studies did not consistently report the timing of blood sample collection (seasonal variation). Collectively, these heterogeneous factors may introduce unknown biases that could adversely impact the reliability of pooled data, and thus, should be considered when interpreting our findings. Moreover, in one study, although the concentration of VD was measured before the initiation of therapy and oral supplementation, and the authors referenced scientific literature demonstrating the ability of supplementation to maintain VD levels consistently above 30 ng/ml in patients with metastatic colon cancer, formal monitoring of VD in their case series was lacking [31].

VD has demonstrated clear anti-carcinogenic properties and the ability to modulate immune responses, which can potentially enhance the efficacy of chemotherapy. Based on the results of our meta-analysis, it is imperative for future studies to investigate whether VD supplementation in this clinical setting could serve as an innovative approach to improve clinical outcomes.

### Supplementary Information

**Additional file 1.** Various methodological aspects and outputs of the quality assessment scales employed in the study.

## Data Availability

The datasets supporting the conclusions of this article are available from the corresponding author on reasonable request.

## Abbreviations

CI: Confidence interval
CRC: Colorectal cancer
EBA: Electrochemiluminescence binding assay
EP: Event probability
HR: Hazard ratio
MINORS: Methodological Index for Non-Randomized Studies
OS: Overall survival
PFS: Progression-free survival
RIA: Radioimmunoassay
RoB2: Risk of Bias 2
VD: Vitamin D
VDR: VD receptor
