# Supplementary material for: Circulating vitamin D level before initiating chemotherapy impacts on the time-to-outcome in metastatic colorectal cancer patients: systematic review and meta-analysis
Source: J Transl Med. 2024 Jan 30;22:119. doi: 10.1186/s12967-024-04889-2 (PMC10826188; doi:10.1186/s12967-024-04889-2)
Supplement: Supplementary file 1 — Additional file 1. Various methodological aspects and outputs of the quality assessment scales employed in the study. [file 12967_2024_4889_MOESM1_ESM.docx]

**Additional File 1**

| **Aspect evaluated** | **MINORS** | **NOS** | **RoB2** |
| --- | --- | --- | --- |
| Study design | Explicit criteria for assessing non-randomized studies | Differentiates between cohort and case-control studies | Appraises the randomization process and study design |
| Control group | Presence and appropriateness of a control group | Assesses the selection and comparability of the control group | Evaluates allocation concealment, blinding, and baseline similarities |
| Baseline comparability | Evaluates baseline comparability between groups | Examines comparability based on characteristics and confounders | Assesses baseline similarities and adjustments |
| Patient selection | Clear description of patient selection process | Criteria for patient selection and representativeness of the sample | Evaluates the recruitment process and eligibility criteria |
| Endpoint assessment | Adequate reporting and evaluation of study endpoints | Assess the outcome measurement and ascertainment of outcomes | Evaluates outcome measurement and reporting |
| Statistical analysis | Considers statistical analysis and power calculation | Considers statistical methods, appropriateness, and power | Examines statistical analysis, bias, and precision |
| Loss to follow-Up | Addresses and accounts for loss to follow-up | Considers loss to follow-up and adequacy of follow-up duration | Evaluates completeness of follow-up and handling of missing data |
| Confounding factors | Assesses the control of confounding factors | Addresses confounding factors and control measures | Examines methods to control confounding and other biases |
| Interventions/Exposures | Evaluates the description and appropriateness | Examines exposure/intervention definition and measurement | Assesses intervention/exposure, randomization, and blinding |
| Quality of reporting | Considers the overall quality of reporting | Examines the clarity and completeness of reporting | Assesses reporting bias and selective outcome reporting |
| Score range (Min - Max) | 0 - 16 | 0 - 9 (for cohort or case-control studies), 0 - 10 (for cohort studies) | 0 - 11 |
| Main aspect described | Assesses the overall methodological quality of non-randomized studies | Focuses on selection, comparability, and outcome assessment for observational studies | Evaluates the risk of bias related to study design |

**Various methodological aspects and outputs of the quality assessment scales employed in the study.**
